# Utility of methylene blue mixed with autologous blood in preoperative localization of pulmonary nodules and masses

**DOI:** 10.1515/biol-2022-0645

**Published:** 2023-07-09

**Authors:** Zhi Feng, Qiu-Xia Liao, Jin-Bao Xie, Jian-Feng Chen, Ming-Lian Qiu, Xu Li

**Affiliations:** Department of Thoracic Surgery, The First Affiliated Hospital of Fujian Medical University, Fuzhou, 350005, Fujian Province, China; Department of Critical medicine, The First Affiliated Hospital of Fujian Medical University, Fuzhou 350005, China; Department of Thoracic Surgery, National Regional Medical Center, Binhai Campus of The First Affiliated Hospital, Fujian Medical University, Fuzhou, 350212, China

**Keywords:** methylene blue, autologous blood, pulmonary nodules and masses, localization

## Abstract

The value of CT-guided puncture with methylene blue mixed with autologous blood in preoperative localization of pulmonary nodules and masses was explored. A total of 113 patients with 146 nodules and masses were treated with methylene blue mixed with autologous blood for preoperative localization and thoracoscopic surgery in the Department of Thoracic Surgery, the First Affiliated Hospital of Fujian Medical University between October 2021 and October 2022. The localization effect, complications, and pathological conditions were observed. The localization success rate was 98.63% (144/146). The localization failed nodules and masses could still be located by looking for needle eyes and reading films. The whole group successfully completed thoracoscopic surgery. The average interval of operation after puncture was 22.16 ± 6.22 h. There was a small amount of suspicious hemothorax after puncture. There was no pneumothorax after puncture in the whole group. There were no hemoptysis, irritating dry cough, and other reactions. The overall complication rate was 2.65%, and no special treatment was given. It is safe and effective to use methylene blue mixed with autologous blood for CT-guided preoperative puncture and localization of small pulmonary nodules and masses.

## 1 Introduction

With the emphasis on lung cancer screening and the widespread application of low-dose spiral CT in physical examination, the number of patients with small pulmonary nodules and masses has been increasing in recent years. Ground-glass nodules have a high probability of malignancy confirmed by pathology, and early surgical intervention has an ideal effect. However, small pulmonary nodules are often insufficient under thoracoscopy and difficult to palpate [1]. Without preoperative localization, it may lead to conversion to thoracotomy or even surgical failure [2].

At present, CT-guided percutaneous lung localization has become a common method of preoperative localization, and a variety of positioning materials have reported good positioning effects, for example, hookwire, microcoil, dye, medical surgical adhesive, or radionuclide. the mean success rate for all techniques of positioning methods in the included relevant studies was 98.1% (94.9–100%), but almost every positioning material has its corresponding advantages and disadvantages [3].

Since October 2021, we have tried to use methylene blue mixed with autologous blood for preoperative CT-guided percutaneous lung localization of small pulmonary nodules and masses in our department, and good results have been achieved.

## 2 Methods and materials

### 2.1 Subjects

A total of 113 patients with pulmonary nodules and masses diagnosed by chest CT in the Department of Thoracic Surgery, the First Affiliated Hospital of Fujian Medical University from October 2021 to October 2022 were collected, including 39 males and 74 females, and the median age of 51.91 ± 11.64 years. Inclusion criteria were as follows: the lesion had no pleural traction sign and no pleural involvement. Exclusion criteria were as follows: pulmonary vascular lesions or lesions close to the pulmonary great vessels and patients with severe cardiopulmonary insufficiency or bleeding tendency. The data of nodules and masses characteristics, diameter, location, localization time, localization complications, the interval between localization and operation, surgical methods, and postoperative pathology were collected. This study was approved by the Ethics Committee of the First Affiliated Hospital of Fujian Medical University (MTCAECFAH of FMU 2015]084-1), and all patients signed “informed consent form.”


**Informed consent:** Informed consent has been obtained from all individuals included in this study.
**Ethical approval:** The research related to human use has been complied with all the relevant national regulations, institutional policies, and in accordance with the tenets of the Helsinki Declaration, and has been approved by Ethics Committee of the First Affiliated Hospital of Fujian Medical University (MTCAECFAH of FMU 2015]084-1).

### 2.2 Method of positioning

Methylene blue mixed with autologous blood was used for preoperative localization of small pulmonary nodules and masses and thoracoscopic surgery. One day before or on the day of surgery, 5–10 ml of peripheral venous blood was drawn and mixed with 1 ml of methylene blue before puncture. After the patient entered the CT room, the appropriate position was determined according to the location of the nodule by reading the film. The 18-G PTC needle (Hakko Co., Ltd, Japan) was gradually inserted 1 cm away from the lesion and then withdrawn to confirm that it did not enter the bronchus or blood vessels. About 3 ml of methylene blue-autologous blood mixture was injected, and the needle was removed at the end of the operation. Postoperative scanning was performed by 64-slice spiral CT scanner (Siemens SOMATOM Definition AS, Germany), to confirm whether the distribution of markers was satisfactory and whether pneumothorax or hemothorax occurred after puncture.

### 2.3 Surgical procedure

**Figure 1:**
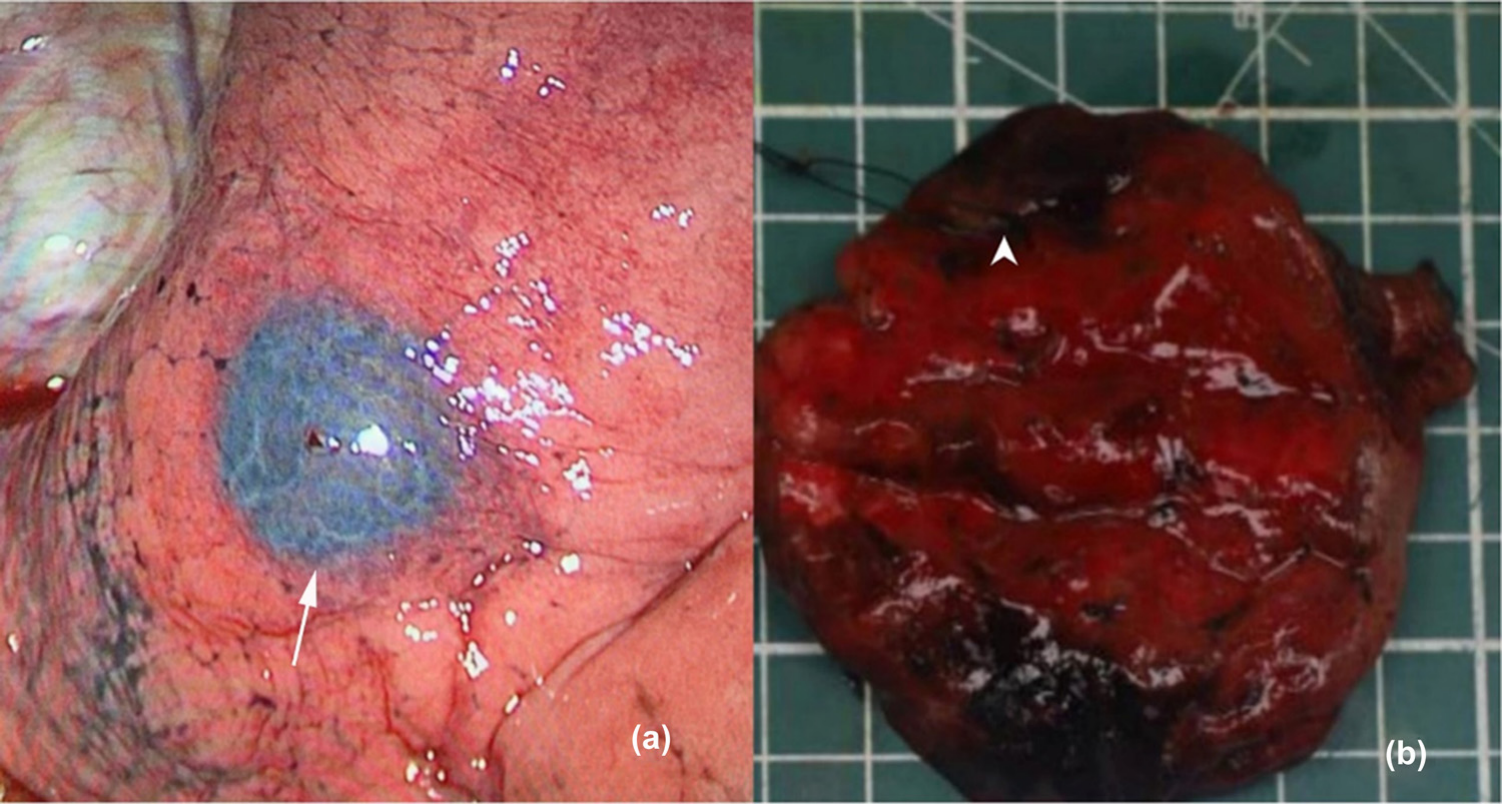
Representative images before and after operation. (a) The markers appeared bule-purple on thoracoscopy (as the white arrow points); (b) after dissection, the gray-white nodules were marked with a line (as the white arrow points).

Patients were generally brought to the operating room 1 day or the same day after localization. All patients underwent single-utility port thoracoscopic surgery with double-lumen endotracheal intubation under general anesthesia and one-lung ventilation. The thoracoscopic hole was made at the midaxillary line of the seventh intercostal space, and the main operation hole was made at the anterior axillary line of the fourth intercostal space. Thoracoscopic exploration was performed, and a dark purple ecchymosis sign was found on the pleural surface of the lung layer (Figure 1), with a clear boundary with the surrounding tissues. For the nodules and masses close to the lung surface, a wedge resection was performed, and a linear cutting closed suture device was used to perform a wedge resection. The specimen was carefully searched according to the puncture path, and the frozen section was sent during the operation to confirm the posterior line. For the deep nodules and masses, segmentectomy was performed, and the segmental arteries and bronchi were treated. The inflation-collapse method was used to confirm the boundary, and the linear cutting closed suture device was used to remove the specimen. After confirming the location of the nodules and masses, the frozen section was sent to determine whether to perform lymph node dissection and lobectomy.

### 2.4 Statistical analysis

Data were analyzed by SPSS 22.0. Data were shown as mean ± standard deviation (SD). Fisher’s precision probability test was used to compare the difference in the probability of pneumothorax and hemothorax after puncture between the groups. A *P*-value of less than 0.05 was considered statistically significant.

## 3 Results

### 3.1 Positioning and analysis of nodules and masses

The clinical characteristics of the patients are shown in Table 1. Single nodule/mass was located in 85 cases, 2 nodules/masses in 23 cases, and 3 nodules/masses in 5 cases. The average diameter of nodules and masses was (8.39 ± 4.00) mm (range 3–31 mm), and the average vertical distance between nodules/masses and pleura was 11.11 ± 8.07 mm (range 0–31.5 mm).

**Table 1:** Clinical data of all patients

Characteristic	Results
Age, years (mean ± SD)	51.91 ± 11.64
Sex (male/female)	39/74
Number of localized nodules and masses	
1	85
2	23
3	5
Characteristics of nodules and masses	
pGGO	113
mGGO	17
Solid	16
Diameter of nodules and masses	
≤1.0 cm	120
＞1.0 cm	26
Location of nodules and masses	
Upper/middle/lower lobes of the right lung	59/6/26
Upper/lower lobes of the left lung	39/18
Time from positioning to surgery, h (mean ± SD)	22.16 ± 6.22

SD, standard deviation; pGGO: pure ground-glass opacity; mGGO: mixed ground glass opacity.

Among 146 nodules and masses, 144 nodules and masses were successfully identified and 2 nodules and masses were failed, with a success rate of 98.63% (144/146). As for the two nodules and masses that failed to be identified, one showed only a small amount of haze formation in the lung tissue after localization, which was not clearly displayed during the operation. The location of the other one was too deep, and the lung itself was seriously pigmented, and no obvious pleural hematoma and purple markers were found by thoracoscopy during operation. It was necessary to confirm the position of the needle eye of the chest wall after lung inflation and to comprehensively determine the location of the nodules and masses.

The remaining 144 localized nodules and masses were identified as typical purplish red pleural hematoma under thoracoscopy. The average time from the detection of nodules and masses to localization was 13.63 ± 5.14 min. The average time from puncture to surgery in 113 patients was about 22.16 ± 6.22 h. After positioning, the stained area of pleura marked with purple red was explored under thoracoscopy during the operation (Figure 1).

### 3.2 Complications

One case had a small amount of suspected hemothorax after puncture, but no progress was found after observation. Intraoperative exploration showed that pleural hemorrhage did not exceed 80 ml. Two cases had a small amount of pneumothorax after puncture, and the compression did not exceed 90%, and no special treatment was given. No hemoptysis, irritating dry cough, thrombosis, and other reactions occurred in the whole group, and the overall complication rate was 2.65%. In addition to the cost of puncture operation, only an additional cost of 12.25 yuan/methylene blue was incurred.

### 3.3 Postoperative pathological diagnosis of nodules and masses

The postoperative pathological diagnosis of 146 nodules and masses was as follows: malignant nodules and masses accounted for 80.82% (118/146), including 5 atypical adenomatous hyperplasia, 58 adenocarcinoma in situ, 42 minimally invasive adenocarcinoma, and 13 invasive adenocarcinoma, and benign nodules/masses accounted for 19.18% (28/146).

### 3.4 Comparison of probability of pneumothorax and hemothorax

The 113 patients were divided into 1 nodule/mass localization group, 2 nodules/masses localization group, and 3 nodules/masses localization group. There was no significant difference in the probability of pneumothorax and hemothorax after puncture between the groups (*P* > 0.05) (Table 2).

**Table 2:** Comparison for incidence of complications between different positioning arrays

	Group with 1 nodule and mass	Group with 2 nodules and masses	Group with 3 nodules and masses	*P*
*N*	85	23	5	—
Pneumothorax	1	1	0	0.436
Hemothorax	0	1	0	0.248

## 4 Discussion

CT-guided percutaneous lung localization is an important auxiliary method for the surgical treatment of small pulmonary nodules and masses. At present, several methods are commonly used: (1) a Hookwire localization method, which is the most common puncture technique [4], is CT-guided percutaneous puncture with a hook wire; the metal wire can be lifted to make the lesion located at the top of the lung tissue, which is easy to grasp the resection margin. VATS surgery should be performed within 1–2 h after puncture. The disadvantage is that the wire is easily displaced and leads to positioning failure, which produces the risk of complications such as pneumothorax, hemoptysis, and pleural reaction, and in addition, the positioning needle will be difficult to handle if the operation is suspended for various reasons. Patients with conditions can choose to perform surgery immediately after puncture in a hybrid operating room, which can effectively reduce the occurrence of complications [5]. (2) The positioning method of percutaneous puncture coil [6], which is a platinum microcoil commonly used for interventional embolization, has no barbed design, and the tip of the microcoil is released around the nodule and the tail is released on the lung surface under the guidance of CT. The localization of the nodules and masses can be accomplished by inspection, palpation, and lifting the tail. Compared with a Hookwire system, the small nodules can be fixed in place by the friction between the spring coil and the lung tissue [7,8]. (3) Percutaneous puncture liquid material injection positioning method, liquid material including lipiodol, methylene blue, indocyanine green, etc. [9,10]. All kinds of positioning materials have their own advantages and disadvantages, and all of them have certain value, but they also have the risk of allergy, pneumothorax, bleeding, and pleural reaction [3,11]. In addition, there are other methods such as intraoperative ultrasound localization, virtual reality technology-assisted localization, and intraoperative electromagnetic navigation bronchial localization. However, due to the complicated steps and high cost, their clinical promotion is limited.

When methylene blue is used for localization alone, the operation should be performed within 2 h after puncture because of the rapid diffusion speed, otherwise the localization will fail due to the diffusion of the marker [12]. Over-injection of methylene blue may also cause diffuse staining and lead to localization failure. The success rate of localization is about 85%, which is lower than that of methods such as Hookwire localization [13]. It has the advantage of less complications and lower cost [14]. In clinical practice, if the operation cannot be performed immediately after puncture, this method is not suitable, so the application of methylene blue in puncture localization is limited. Mixing with other liquid positioning materials is also a scheme to improve the positioning effect of methylene blue puncture. If it is mixed with medical tissue glue for positioning, its diffusion can be avoided and better positioning effect can be achieved. However, tissue glue is a foreign body, which may cause irritating cough. In addition, the glue and methylene blue may coagulation during the mixing process so as to affect the use. It is necessary to explore the ratio of glue produced by different manufacturers and methylene blue that may be applicable [15,16].

When using autologous blood alone, the hematoma formed by autologous blood puncture also has the problem of diffusion. Generally, it is recommended to complete the operation within 12 h, otherwise the hematoma may disappear [17]. In addition, there is the problem of insufficient color development, especially for patients with severe pigmentation on the surface of the lung itself; it may be unclear and lead to localization failure. However, this method also has many advantages, because autologous blood injection through the needle tract itself can reduce the incidence of pneumothorax during lung puncture [18], and there are also reports that autologous blood is used as a patch to treat refractory pneumothorax [19]. The probability of pneumothorax and hemothorax is lower than other puncture methods, and it also has the advantages of convenient sampling, small rejection reaction, and self-absorption of hematoma even if the operation cannot be performed. If improved, it is expected to become a safer and more reliable new localization method.

The use of methylene blue combined with autologous blood for preoperative puncture localization overcomes the shortcomings of insufficient diffusion absorption and color development of the above two materials, which not only improves the lack of color development but also prolongs the diffusion absorption time. The localization success rate was 98.63% (144/146), which was comparable with other methods (successful targeting rates [95% confidence interval] for hookwire, microcoil, and lipiodol localization were 0.98 (0.97, 0.99), 0.98 (0.96, 0.99), and 0.99 (0.98, 1.00), respectively) [20]. In this study, the average interval between puncture localization and operation was 22.16 ± 6.22 h, and the operation was performed almost 1 day after the completion of localization. The overall satisfactory positioning effect was still maintained. This method is more flexible than using methylene blue or autologous blood alone, and it can be used to locate the tumor 1 day before the operation. When methylene blue mixed with autologous blood is used for localization, the formation of subpleural blue-purple hematoma can be seen during the operation, which is easier to identify than autologous blood alone, and plays a good role in identifying the location.

This method has few complications (2.65%), no serious complications were found in this study, and no special treatment was given after puncture positioning to the operation. There was no obvious pneumothorax in the whole group, and the major complications rate of our method was also lower than hookwire, microcoil, medical dye, medical surgical adhesive, and contrast agent location [3]. The incidence of pneumothorax was lower than using autologous blood puncture positioning [17], which was related to the use of thinner PTC needle (18G).

The advantage of a liquid-based locator is that it allows the use of a finer needle than a metallic locator. Although a thinner puncture needle may reduce the risk of puncture trauma and pneumothorax, the finer the puncture needle is not the better. If the location is not obvious, a smaller puncture point is more likely to lead to difficulty in finding the puncture point, which may increase the risk of surgical failure.

Both methylene blue and autologous blood have great advantages in terms of the availability and economy of the required materials. Methylene blue as a simple routine preparation is in sufficient supply, and the acquisition of autologous blood is not affected by the supply chain of drug consumables. In addition, under the background of the gradual promotion of DRG payment mode of medical insurance, the more economical puncture positioning scheme also makes the total hospitalization cost of patients not easy to exceed the standard.

In summary, the use of methylene blue mixed with autologous blood for CT-guided lung puncture positioning has the advantages of high accuracy, low incidence of complications, relatively simple positioning operation, low cost, and easy-to-obtain required consumables. However, there are still disadvantages, such as poor identification of cases with severe lung surface pigmentation, and unclear display when puncture is deep from the pleura. The overall effect of this method is satisfactory and can be carried out in most hospitals, which is worthy of further clinical promotion.
